# Three-dimensional alignment of the upper extremity in the standing neutral position in healthy subjects

**DOI:** 10.1186/s13018-022-03113-5

**Published:** 2022-04-15

**Authors:** Yuki Yoshida, Noboru Matsumura, Yoshitake Yamada, Satoshi Hiraga, Kazunori Ishii, Satoshi Oki, Yoichi Yokoyama, Minoru Yamada, Masaya Nakamura, Takeo Nagura, Masahiro Jinzaki

**Affiliations:** 1 Department of Orthopedic Surgery, Keio University School of Medicine, 35 Shinanomachi, Shinjuku-ku, Tokyo, 160-8582 Japan; 2 Department of Radiology, Keio University School of Medicine, 35 Shinanomachi, Shinjuku-ku, Tokyo, 160-8582 Japan

**Keywords:** Neutral posture, Shoulder, Elbow, Scapulothoracic joint, Glenohumeral joint, Upright computed tomography

## Abstract

**Background:**

Though alignment of the spine and lower extremities in the standing neutral position has been evaluated, a few studies evaluating the alignment of the upper extremities have also been made. This study assessed the normal alignment of the upper extremities in the standing neutral position and clarified the three-dimensional angular rotations of the upper extremity joints.

**Methods:**

Computed tomography (CT) images of 158 upper extremities from 79 healthy volunteers were prospectively acquired in the standing neutral position using an upright CT scanner. Three-dimensional coordinate systems of the thorax, scapula, humerus, and forearm were designated, and three-dimensional angular rotations of the scapulothoracic, glenohumeral, and elbow joints were calculated.

**Results:**

The median angle of the scapulothoracic joint was 9.2° (interquartile range [IQR], 5.2°–12.5°) of upward rotation, 29.0° (IQR, 24.9°–33.3°) of internal rotation, and 7.9° (IQR, 4.3°–11.8°) of anterior tilt. The median angle of the glenohumeral joint was 4.5° (IQR, 0.9°–7.8°) of abduction, 9.0° (IQR, 2.2°–19.0°) of internal rotation, and 0.3° (IQR, − 2.6°–3.1°) of extension. The median angle of the elbow joint was 9.8° (IQR, 6.9°–12.4°) of valgus, 90.2° (IQR, 79.6°–99.4°) of pronation, and 15.5° (IQR, 13.2°–18.1°) of flexion. Correlations in angular rotation values were found between the right and left upper extremities and between joints.

**Conclusions:**

This study clarified the three-dimensional angular rotation of upper extremity joints in the standing neutral position using an upright CT scanner. Our results may provide important insights for the functional evaluation of upper extremity alignment.

## Background

Human posture is generally viewed as the coordination among the parts of the human body in the standing neutral position. The neutral standing position of the human body is described according to the following alignment: the frontal view is symmetrical; from the lateral view, a perpendicular line begins at the mastoid process of the temporal bone and runs vertically through the acromion, lumbar vertebral bodies, and greater trochanter (slightly posterior to the hip axis and slightly anterior to the knee axis) and ends at the lateral malleolus or slightly anterior to it [1]. The course of this line in normal neutral standing position overlaps the baseline between the center of gravity and the center point of the support [1, 2]. On the other hand, the neutral position of the upper extremities is defined as the arms resting at the side with the shoulders in neutral rotation and the palms aligned with the body trunk. This position has been regarded as the standard upper extremity position in previous motion analyses [3–5] and radiographic imaging studies [6, 7].

It is clinically useful to understand the alignment of the standing neutral position to evaluate an altered joint position or decide a treatment plan for deformities. Although numerous studies have investigated the neutral position of loaded joints, such as the spine and the lower extremities [8–10], a few studies have evaluated the neutral posture of the upper extremities, those have assessed only the scapula position [11, 12]. In addition, the evaluation was performed on the skin and might not accurately capture the three-dimensional (3D) position of the bone. In assessing bone position, there have been studies on the sagittal alignment of whole axial skeletons using radiographs [13], although the upper extremities in the sagittal plane cannot be evaluated due to overlapping into the trunk of the arms when placed in the hands-on-cheeks position. Conventional computed tomography (CT) that allows for accurate 3D bone assessment could only be conducted in the supine position. To the best of our knowledge, no detailed study has evaluated the 3D alignment of the upper extremities in the standing neutral position.

Recently, an upright CT scanner, whose physical characteristics are comparable to those of a conventional CT machine, has been developed and enables 3D whole-torso cross-sectional scanning in the standing position [14]. We hypothesized that interactions of the upper extremity joints are employed to maintain a neutral position, just as they are employed in the spine and lower extremities and that 3D alignment of the upper extremity is well correlated to the contralateral side. The purpose of this study was to evaluate normal 3D alignment of the upper extremities and their interactions in the standing neutral position using the upright CT scanner and to clarify the correlation of 3D angular rotation between the right and left upper extremity joints.

## Methods

### Participants

This prospective study was approved by the institutional review board of Keio University School of Medicine, and written consent was obtained from all the participants (study protocol: #20160384). All experiments were performed in accordance with relevant guidelines and regulations. The prospective subjects included 108 healthy Japanese volunteers with no past illnesses or injuries to the upper extremities, aged between 30 and 60 years. All provided informed consent and agreed to participate in this study and undergo upright CT (prototype TSX-401R; Canon Medical Systems Corporation, Otawara, Japan) [15, 16]. Twenty-three participants were excluded because their distal humeri were out of the range of the CT images. In addition, six participants were excluded because of asymptomatic scoliosis. Thus, CT images of 158 upper extremities from 79 healthy volunteers (25 male and 54 female) were included in the analysis. The mean (± standard deviation) age, height, body weight, and body mass index (BMI) of the subjects were 44.4 ± 7.3 years (range 30–58 years), 161.6 ± 8.3 cm (range 147.7–182.1 cm), 56.3 ± 8.6 kg (range 37.8–79.4 kg), and 21.5 ± 2.8 kg/m^2^ (range 15.7–29.2 kg/m^2^), respectively.

### Image acquisition

**Fig. 1:**
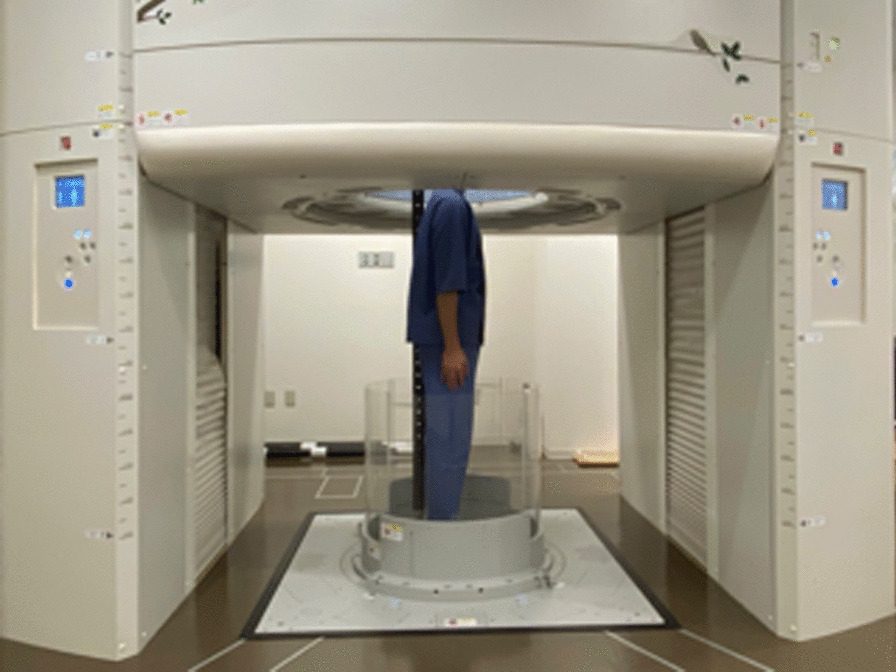
Computed tomography (CT) images of the bilateral upper extremities were obtained in the standing neutral position using an upright CT scanner

The CT images acquired were taken from neck to pelvis using an upright CT scanner in the standing neutral position (Fig. 1). During acquisition, the volunteers were instructed to stand in the relaxed neutral position: spreading their legs according to their shoulder width and touching their sacrum to the pole behind them to keep their body trunk perpendicular to the ground to achieve a safe scanning condition. The upper extremities were positioned with the palms touching the lateral sides of the thighs in a comfortable resting position, defined as the neutral position. Scanning was performed at 100 or 120 kVp and at a gantry rotation speed of 0.5 s in the helical scan mode (80-detector row) with a noise index of 24 or 15 and helical pitch of 0.8 for the body trunk. Image reconstruction was performed using Adaptive Iterative Dose Reduction in 3D (Canon Medical Systems Corporation, Otawara, Japan), which could reduce imaging radiation [17]. The CT data were accumulated in Digital Imaging and Communication in Medicine (DICOM) data format.

### Identification of bony landmarks

**Fig. 2:**
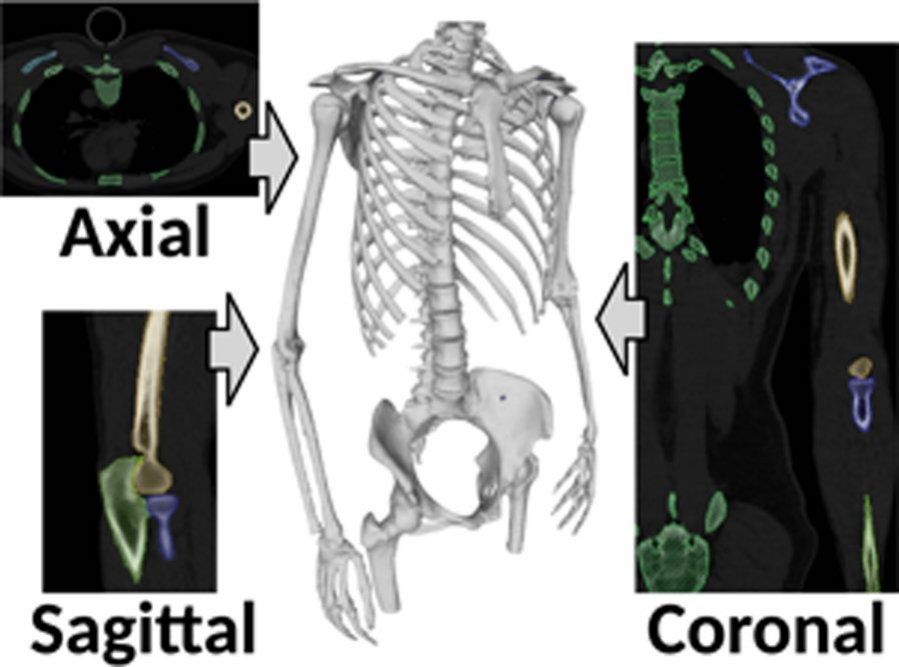
Creating three-dimensional surface models of the body and upper extremities from Digital Imaging and Communication in Medicine data using AVIZO software. Bone part segmentation was performed to observe three views of multiplanar reformatting

**Fig. 3:**
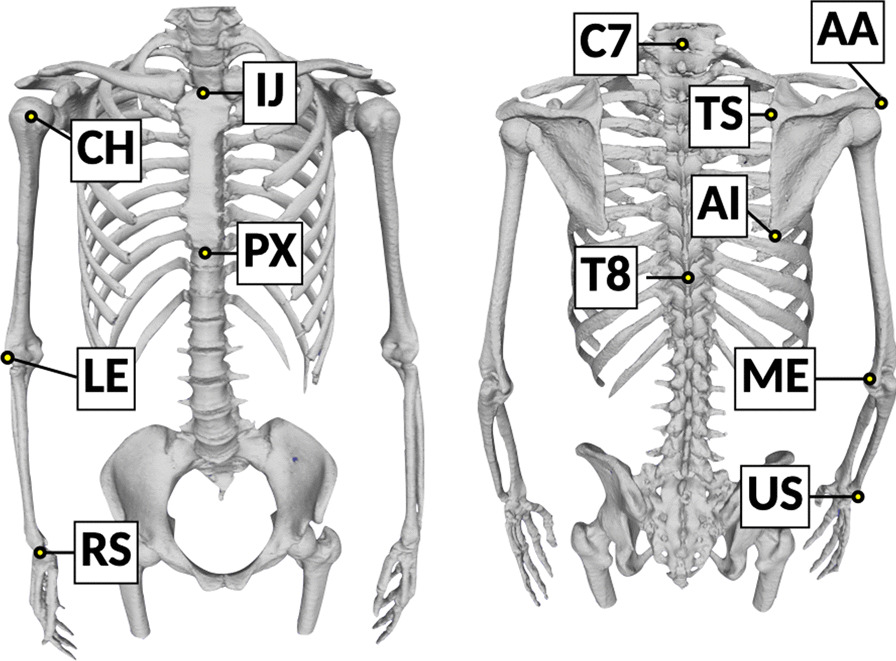
Bony landmarks to create coordinate system of each bone. IJ, sternal notch (incisura jugularis); PX, xiphoid process (processus xiphoideus); C7, spinal process of the 7th cervical vertebra; T8, spinal process of the 8th thoracic vertebra; AA, acromial angle (angulus acromialis); TS, root of the scapular spine (trigonum spinae scapulae); AI, inferior angle (angulus inferior); CH, center of the humeral head; LE, lateral epicondyle; ME, medial epicondyle; RS, radial styloid; US, ulnar styloid

Three-dimensional bone surface models of the trunk and upper extremities were segmented from the DICOM data using AVIZO software (version 9.3.0; Thermo Fisher Scientific, Tokyo, Japan) and exported as Standard Triangulated Language (STL) data (Fig. 2) as previously described [18, 19]. The bony landmarks determined according to the International Society of Biomechanics (ISB) recommendations [20] were identified on STL data of 3D bone surface models using Meshlab software (version 1.3.3; Institute of Information Science and Technologies, Pisa, Italy) (Fig. 3). As the glenohumeral rotation center could not be identified in the static images, the origin of the humeral axis was defined as the center of the humeral head [21, 22].

### Coordinate system

**Fig. 4:**
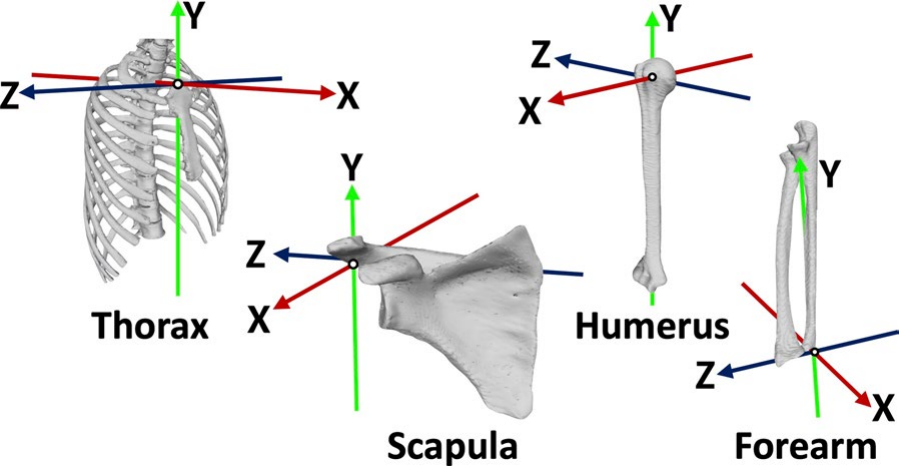
The three-dimensional coordinate system of the thorax, scapula, humerus, and forearm

A coordinate system was designated for each model of the thorax, scapula, humerus, and forearm based on the bony landmarks, as defined by the ISB recommendations [20] (Fig. 4). The left side of the upper extremities was flipped to the right side on the software before designating the coordinate system.

The thoracic coordinate system was defined as follows: The origin was coincident with the deepest point of the sternal notch (incisura jugularis; IJ). The *Y*-axis was defined as the line connecting the midpoint between the xiphoid process (processus xiphoideus) (PX) and the spinal process of the 8th thoracic vertebra (T8) and the midpoint between the IJ and spinal process of the 7th cervical vertebra (C7), pointing upward. The *Z*-axis was the line perpendicular to the plane formed by the IJ, C7, and midpoint between PX and T8, pointing to the right. The *X*-axis was the common line perpendicular to the *Z*- and *Y*-axis, pointing forward.

The scapular coordinate system was defined as follows: The origin was coincident with the acromial angle (angulus acromialis) (AA). The *Z*-axis was defined as the line connecting the root of the scapular spine (trigonum spinae scapulae) (TS), and AA, pointing to the right. The *X*-axis was the line perpendicular to the plane formed by the inferior angle (angulus inferior) (AI), AA, and TS, pointing forward. The *Y*-axis was the common line perpendicular to the *X*- and *Z*-axis, pointing upward.

The humerus coordinate system was defined as follows: The origin was coincident with center of the humeral head (CH) using a sphere fit based on the convex articulating surface above the anatomical neck [21, 22]. The *Y*-axis was defined as the line connecting the CH and the midpoint of the most caudal point on the lateral epicondyle (LE) and medial epicondyle (ME), pointing to CH, and pointing proximally. The *X*-axis was the line perpendicular to the plane formed by the LE, ME, and CH, pointing forward. The *Z*-axis was the common line perpendicular to the *X*- and *Y*-axis, pointing to the right.

The forearm coordinate system was defined as follows: The origin was coincident with the ulnar styloid (US). The *Y*-axis was defined as the line connecting the US and the midpoint between the LE and ME, pointing proximally. The *X*-axis was the line perpendicular to the plane through the US, radial styloid (RS), and the midpoint between the LE and ME, pointing forward. The *Z*-axis was the common line perpendicular to the *X*- and *Y*-axis, pointing to the right.

### Calculations of upper extremity rotation angles

The angular rotations were calculated using the Cardan and Euler angles based on the coordinate system of each bone. The angular rotation of the scapula with regard to the thorax was defined as the scapulothoracic joint angle. The description of the scapulothoracic joint angle was made using the ISB recommended *Y*–*X*–*Z* sequence. The rotation angle was described as the upward/downward rotation about the *X*-axis, internal/external rotation about the *Y*-axis, and anterior/posterior tilt about the *Z*-axis.

The angular rotation of the humerus with regard to the scapula was defined as the glenohumeral joint angle. The ISB recommended *Y*–*X*–*Y* sequence, which has been routinely performed during arm elevation, was not valid for evaluating humeral axial rotation with the arm at the side with a risk of excluding singular positions [23]. Therefore, the rotation sequence had to be changed in agreement with the no-gimbal lock incidence and amplitude interpretability of the performed movements [24]. In this study, the description of the glenohumeral joint angle was made using the *X*–*Z*–*Y* sequence, which is reported to have greater clinical applicability than the *Y*–*X*–*Y* sequence [25]. Rotation angle was described as abduction/adduction about the *X*-axis, internal/external rotation about the *Y*-axis, and flexion/extension about the *Z*-axis [24].

The angular rotation of the forearm with regard to the humerus was defined as the elbow joint angle. The description of the elbow joint angle was made using the ISB recommended *Z*–*X*–*Y* sequence. The rotation angle was described as valgus/varus about the *X*-axis, pronation/supination about the *Y*-axis, and flexion/extension about the *Z*-axis.

### Statistical analysis

SPSS Statistics 27.0.1.0 software (IBM Corp., Armonk, NY, USA) was used for statistical analyses. The intraobserver and interobserver reliabilities for the angular rotation values were assessed by calculating intraclass correlation coefficients (ICCs) based on randomly selected 20 upper extremities. The measurements were made blindly by two observers (ICC model 2,1), and repeated measurements by one observer within a 3-month interval (ICC model 1,1). After determining intra- and interrater reliabilities, the assessments were performed for all subjects by one observer.

The rotation angle data partly presented a non-normal distribution using the Shapiro–Wilk test, and nonparametric tests were used in the analyses. Descriptive statistics of these data were presented using median and interquartile range (IQR). The differences between male and female participants were assessed using Mann–Whitney *U* tests. The correlations in angular rotation values between the right and left upper extremities and the relationships between the angular rotation values of each joint in the *X*, *Y*, and *Z* axes were evaluated using Spearman’s rank correlation coefficient. The significance level was set at *P* < 0.05 for all analyses.

## Results

The intraobserver and interobserver correlation coefficients for the angular rotation values exceeded 0.85 for all calculations (Table 1). These results confirmed that the angular rotation values were highly reproducible.

**Table 1:** Participant characteristics and three-dimensional angular rotation values of the scapulothoracic, glenohumeral, and elbow joints

	Mean ± SD			Sex difference	ICC (95% CI)	
	Median (IQR)					
	Total	Male	Female	*P* value	Intrarater	Interrater
Parameters						
Age, years old	44.4 ± 7.3	44.8 ± 7.6	44.2 ± 7.2	0.731		
Height (cm)	161.6 ± 8.3	170.8 ± 5.9	157.3 ± 5.3	< 0.001*		
Weight (kg)	56.3 ± 9.1	64.7 ± 7.5	52.3 ± 6.9	< 0.001*		
BMI (kg/m^2^)	21.5 ± 2.8	22.2 ± 2.7	21.2 ± 2.8	0.003*		
Scapulothoracic joint						
Upward rotation (°)	9.2 (5.2–12.5)	8.6 (5.0–11.8)	9.4 (5.3–12.9)	0.547	0.999 (0.997–1.000)	0.994 (0.957–0.999)
Internal rotation (°)	29.0 (24.9–33.3)	29.3 (24.8–32.6)	28.7 (24.8–33.6)	0.820	0.998 (0.994–1.000)	0.999 (0.993–1.000)
Anterior tilt (°)	7.9 (4.3–11.8)	10.7 (5.6–14.1)	7.0 (3.3–10.2)	< 0.001*	0.996 (0.984–0.999)	0.926 (0.630–0.983)
Glenohumeral joint						
Abduction (°)	4.5 (0.9–7.8)	3.1 (0.0–6.9)	4.9 (1.9–8.5)	0.068	0.998 (0.994–0.999)	0.982 (0.957–0.993)
Internal rotation (°)	9.0 (2.2–19.0)	9.8 (2.4–16.3)	8.5 (1.0–19.2)	0.761	0.953 (0.888–0.981)	0.953 (0.888–0.981)
Extension (°)	0.3 (− 2.6–3.1)	− 0.4 (− 3.3–2.6)	0.6 (− 2.1–3.6)	0.148	0.986 (0.967–0.995)	0.923 (0.819–0.969)
Elbow joint						
Valgus (°)	9.8 (6.9–12.4)	8.1 (5.3–10.5)	10.4 (7.8–13.1)	< 0.001*	0.972 (0.931–0.989)	0.854 (0.668–0.939)
Pronation (°)	90.2 (79.6–99.4)	86.7 (78.0–98.3)	90.8 (81.4–99.4)	0.259	0.938 (0.852–0.975)	0.938 (0.823–0.976)
Flexion (°)	15.5 (13.2–18.1)	16.1 (13.6–18.7)	15.2 (12.8–17.3)	0.123	0.986 (0.967–0.995)	0.834 (0.630–0.931)

*SD* standard deviation, *ICC* intraclass correlation coefficient, *CI* confidence interval

*P < 0.05

The median angle of the scapulothoracic joint was 9.2° (IQR, 5.2°–12.5°) of upward rotation, 29.0° (IQR, 24.9°–33.3°) of internal rotation, and 7.9° (IQR, 4.3°–11.8°) of anterior tilt. The median angle of the glenohumeral joint was 4.5° (IQR, 0.9°–7.8°) of abduction, 9.0° (IQR, 2.2°–19.0°) of internal rotation, and 0.3° (IQR, − 2.6°–3.1°) of extension. The median angle of the elbow joint was 9.8° (IQR, 6.9°–12.4°) of valgus, 90.2° (IQR, 79.6°–99.4°) of pronation, and 15.5° (IQR, 13.2°–18.1°) of flexion.

**Fig. 5:**
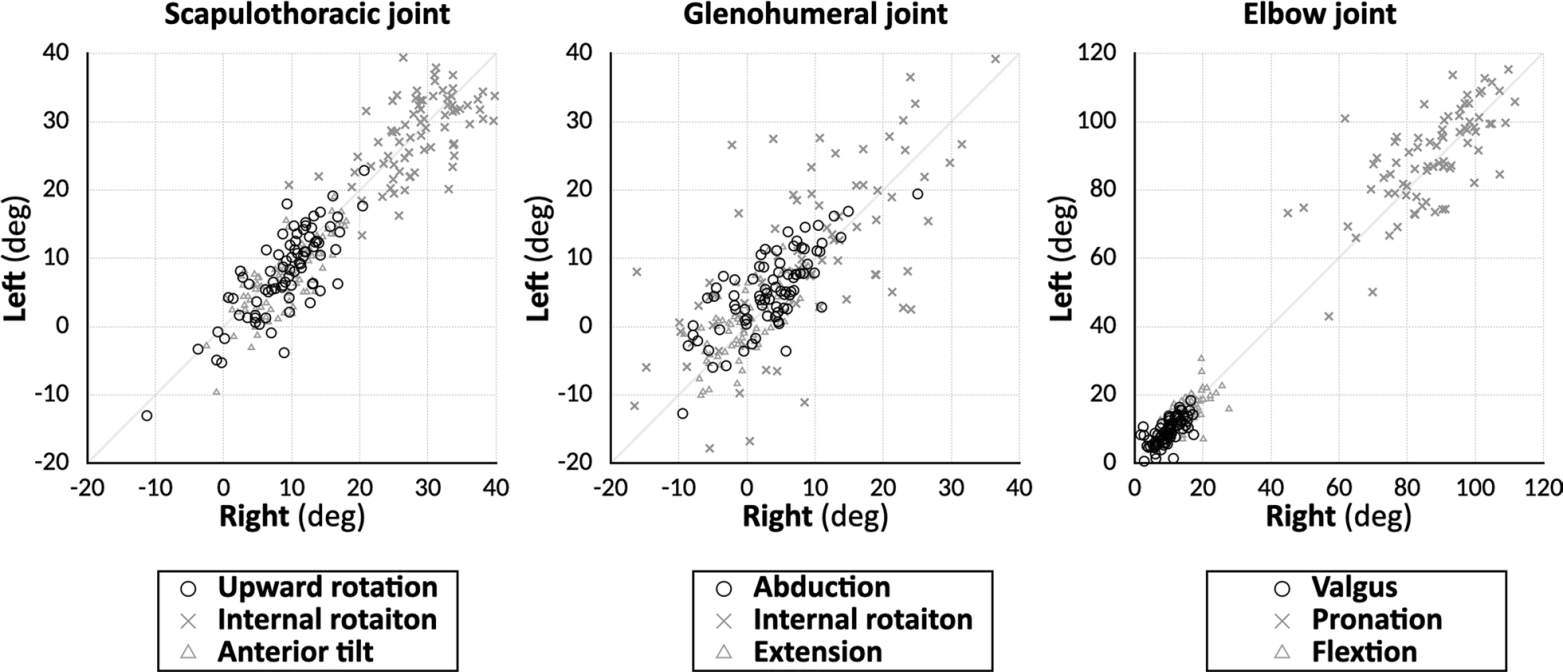
Linear regression plots of angular rotation values compared between the right and left upper extremities. Values showed a strong positive correlation in all angles (scapulothoracic joint: upward rotation *ρ* = 0.743, *P* < 0.001; internal rotation *ρ* = 0.553, *P* < 0.001; anterior tilt *ρ* = 0.740, *P* < 0.001; glenohumeral joint: abduction *ρ* = 0.700, *P* < 0.001; internal rotation *ρ* = 0.595; *P* < 0.001; extension *ρ* = 0.641, *P* < 0.001. elbow joint: valgus *ρ* = 0.736, *P* < 0.001; pronation *ρ* = 0.702, *P* < 0.001; and flexion *ρ* = 0.623, *P* < 0.001)

Significant differences were observed between males and females not only in the physique (height, weight, and BMI) but also in the angular rotation values of the anterior tilt and the valgus. The anterior tilt of the scapulothoracic joint was significantly greater in males (10.7° [IQR, 5.6°–14.1°]) than in females (7.0° [IQR, 3.3°–10.2°], *P* < 0.001). The valgus of the elbow joint was significantly greater in females (10.4° [IQR, 7.8°–13.1°]) than in males (8.1° [IQR, 5.3°–10.5°, *P* < 0.001) (Table 1). The right and left upper extremities’ angular rotation values showed strong positive correlations at all angles (Fig. 5).

In the *X*-axis angular rotation, there was a strong negative correlation between the upward rotation of the scapulothoracic joint and the abduction of the glenohumeral joint (*ρ* = − 0.545, *P* < 0.001) (Table 2). In the *Y*-axis angular rotation, there was a weak negative correlation between the pronation of the elbow joint and the internal rotation of the glenohumeral joint (*ρ* = − 0.337, *P* < 0.001) (Table 3). In the *Z*-axis angular rotation, there was a moderate negative correlation between the anterior tilt of the scapulothoracic joint and extension of the glenohumeral joint (*ρ* = − 0.506, *P* < 0.001) (Table 4).

**Table 2:** Correlation between the angular rotation values of each joint in the *X*-axis

*X*-axis angular rotation	Pearson’s correlation coefficient	Scapulothoracic upward rotation	Glenohumeral abduction	Elbow valgus
Scapulothoracic upward rotation	*ρ* (*P* value)		− 0.545 (< 0.001*)	− 0.116 (0.146)
Glenohumeral abduction	*ρ* (*P* value)	− 0.545 (< 0.001*)		0.017 (0.892)
Elbow valgus	*ρ* (*P* value)	− 0.116 (0.146)	0.017 (0.892)	

*P < 0.05

**Table 3:** Correlation between the angular rotation values of each joint in the *Y*-axis

*Y*-axis angular rotation	Pearson’s correlation coefficient	Scapulothoracic internal rotation	Glenohumeral internal rotation	Elbow pronation
Scapulothoracic internal rotation	*ρ* (*P* value)		− 0.056 (0.481)	0.007 (0.927)
Glenohumeral internal rotation	*ρ* (*P* value)	− 0.056 (0.481)		− 0.337 (< 0.001*)
Elbow pronation	*ρ* (*P* value)	0.007 (0.927)	− 0.337 (< 0.001*)	

*P < 0.05

**Table 4:** Correlation between the angular rotation values of each joint in the *Z*-axis

*Z*-axis angular rotation	Pearson’s correlation coefficient	Scapulothoracic anterior tilt	Glenohumeral extension	Elbow flexion
Scapulothoracic anterior tilt	*ρ* (*P* value)		− 0.506 (< 0.001*)	0.066 (0.413)
Glenohumeral extension	*ρ* (*P* value)	− 0.506 (< 0.001*)		0.277 (< 0.001*)
Elbow flexion	*ρ* (*P* value)	0.066 (0.413)	0.277 (< 0.001*)	

*P < 0.05

## Discussion

The present study revealed the 3D alignment of the upper extremities in the standing neutral position in healthy subjects using an upright CT scanner. To our best knowledge, this is the first study specifically to address the alignment of the upper extremities and the normal values of the rotation of upper extremity joints in the standing neutral position and their interactions. We found the angular rotations of the upper extremity in the standing position were well correlated to those of the contralateral side. Our results could be useful in evaluating and treating pathological abnormalities in the upper extremities.

The angular rotation values in the right and left upper extremities were strongly correlated and generally consistent with one another. When diagnosing an altered joint position or deciding a treatment plan for malunion deformities, contralateral intact extremity is often referenced. In the assessment of the scapular dyskinesis, the altered scapular positioning and motion is associated with shoulder injuries [26] and the alignment change is compared with the contralateral scapula [11]. As for malunion, cubitus varus or valgus deformity is one of the most common complications after fractures in the upper extremity and is treated with reference to the contralateral intact joint [27, 28]. Although comparisons of morphological symmetry [21, 29, 30] or asymmetry [22, 31, 32] between right and left have been reported, the correlation between right and left 3D alignment of the upper extremities has not been evaluated. The present results support the assumption that the right and left joint angles are comparable and suggest that it is reasonable to use the intact joint angle as a reference.

Significant sex differences in angular rotation values were observed in the anterior tilt of the scapulothoracic joint and in the valgus of the elbow joint. There are sex differences in the composition and function of skeletal muscle [33], and the muscles that stabilize the scapula to the thorax, namely the rhomboids, levator scapulae, and trapezius, are stronger in males than in females and can be presumed to support the scapula in an upward direction [34, 35]. Valgus of the elbow joint, which is called the carrying angle, was reported to vary between sex [36, 37], and the present results are consistent with the literature. Concerning the age of the epiphyseal closure around the elbow, there is an increase in the carrying angle until 15 years age. The sex difference in the carrying angle was reported to depend on the joint laxity or the onset of adolescence [37, 38].

**Fig. 6:**
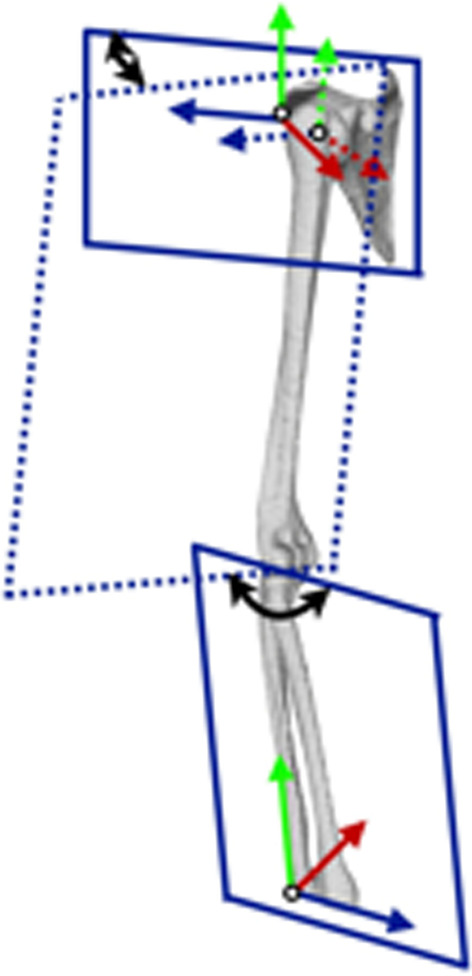
Angular rotation between the pronation of the elbow joint and internal rotation of the glenohumeral joint. There was an interaction for greater pronation to become external rotation

Interactions to maintain balance have been reported in the spine and lower extremities [10, 39, 40], whereas none have been reported in the upper extremities. In this study, we found that these interactions are also observed in the neutral position of the upper extremities. As pelvic tilt correlates with knee flexion to keep the sagittal balance in the neutral position of the lower extremity, internal rotation of the glenohumeral joint correlates with the pronation of the elbow joint to keep the axial balance in the upper extremities. When the internal rotation angle of the glenohumeral joint was decreased, there was a compensatory increase in the pronation of the elbow joint in order to align the palms with the body trunk (Fig. 6). Therefore, in shoulder X-ray imaging, which is mainly taken in a standing neutral position, the positional relationship between the scapula and humerus is often different even if the images are taken in the same posture [6].

These interactions were also observed in the scapulothoracic joint and the glenohumeral joint. In the neutral position of the upper extremities with the arms resting at the side, the glenohumeral joint is relatively adducted when the scapula rotates upward. Similarly, the glenohumeral joint is relatively flexed when the scapula tilts anteriorly. The abduction and extension angle of the glenohumeral joint changes depending on the individual alterations in the resting position of the scapula.

This study had limitations. First, the postural sway in a standing neutral position was not evaluated. However, volunteers were positioned along the pole to avoid any effect of posture. We made sure that the volunteers were in the same position and excluded those with scoliosis. Second, asymptomatic scapular dyskinesis and humeral head retroversion were not evaluated. Although these factors might affect the results of this study, scapular positional and humeral head anatomical variations have been reported to exist among healthy subjects [11, 41, 42], and these differences were included as normal joint angular rotation values. Third, anatomical changes in upper extremity alignment in the pathological state are still unclear because this study evaluated healthy volunteers. It is expected that future studies will clarify the changes of alignment in upper extremity disorders, which will be useful for diagnosis, treatment, and functional assessment.

## Conclusions

This study evaluated the 3D angular rotation of upper extremity joints in the standing neutral position using an upright CT scanner. The angular rotation values in the right and left upper extremities were strongly correlated in all angles, and significant sex differences were observed in the anterior tilt of the scapulothoracic joint and the valgus of the elbow joint. Our results may provide important clues for evaluating and understanding the function of upper extremity alignment.

## Data Availability

The datasets used and/or analyzed during the current study are available from the corresponding author on reasonable request.

## Abbreviations

AA: Acromial angle (angulus acromialis)
AI: Inferior angle (angulus inferior)
BMI: Body mass index
CH: Center of the humeral head
CT: Computed tomography
C7: Spinal process of the 7th cervical vertebra
DICOM: Digital Imaging and Communication in Medicine
ICCs: Intraclass correlation coefficients
IJ: Sternal notch (incisura jugularis)
IQR: Interquartile range
ISB: International Society of Biomechanics
LE: Lateral epicondyle
ME: Medial epicondyle
PX: Xiphoid process (processus xiphoideus)
RS: Radial styloid
STL: Standard Triangulated Language
TS: Root of the scapular spine (trigonum spinae scapulae)
T8: Spinal process of the 8th thoracic vertebra
US: Ulnar styloid
3D: Three-dimensional
